# Toward a Pragmatic Multidisciplinary Management of Nutritional Risk in Hospitalized Patients: Initiatives and Proposals of the Clinical Nutrition Network of Lombardy Region

**DOI:** 10.3390/nu17091472

**Published:** 2025-04-27

**Authors:** Elisa Mattavelli, Elvira Verduci, Annalisa Mascheroni, Ettore Corradi, Valentina Da Prat, Emanuela Ammoni, Danilo Cereda, Alessandro Scardoni, Alessandro Amorosi, Riccardo Caccialanza

**Affiliations:** 1 Clinical Nutrition and Dietetics Unit, Fondazione IRCCS Policlinico San Matteo, 27100 Pavia, Italy; e.mattavelli@smatteo.pv.it (E.M.); v.daprat@smatteo.pv.it (V.D.P.); 2 Metabolic Disease Unit, Department of Paediatrics, Vittore Buzzi Children’s Hospital, University of Milan, 20154 Milan, Italy; elvira.verduci@unimi.it; 3 Department of Health Sciences, University of Milan, 20142 Milano, Italy; 4 Clinical Nutrition and Dietetics Unit, ASST Melegnano e Martesana, 20077 Melegnano, Italy; annalisa.mascheroni@asst-melegnano-martesana.it; 5 Clinical Nutritional Unit, ASST Grande Ospedale Metropolitano Niguarda, 20162 Milano, Italy; ettore.corradi@ospedaleniguarda.it; 6 Welfare General Directorate, Regione Lombardia, 20124 Milano, Italy; emanuela_ammoni@regione.lombardia.it (E.A.); danilo_cereda@regione.lombardia.it (D.C.); alessandro_scardoni@regione.lombardia.it (A.S.); alessandro_amorosi@regione.lombardia.it (A.A.)

**Keywords:** malnutrition, clinical nutrition, nutritional screening, multidisciplinary approach, nutritional risk management

## Abstract

Malnutrition is a widespread problem in hospitalized patients, which significantly impacts clinical outcomes, quality of life, and healthcare costs. Despite its well-documented consequences, it remains underdiagnosed and inadequately managed in many healthcare settings. Even with recent progress, key challenges remain, including inconsistent use of standardized nutritional screening tools and practices, insufficient professional training, and resource limitations. A multidisciplinary approach involving physicians, dietitians, nurses, and pharmacists is crucial for early detection, timely intervention, and prevention of malnutrition-related complications. The sustainability of a multidisciplinary model requires overcoming logistical and financial barriers, including the integration of technology for real-time monitoring, standardized screening protocols, and specific professional training. Regional initiatives, such as the establishment of the Clinical Nutrition Network of Lombardy (Italy), reported and discussed in this article, have made strides in improving nutritional care by promoting scientific networking and standardized practices across hospitals. This approach may not only improve patient outcomes but also reduce long-term healthcare costs by shortening hospital stays and preventing readmissions. For this model to be effective and sustainable, collaboration among healthcare providers, policymakers, and researchers is essential to promote an integrated, cost-effective approach to managing nutritional risk throughout the continuum of care.

## 1. Introduction

The landscape of medicine has evolved significantly over the years, driven by constant advances in research, technology, and treatment modalities. Among these progressive changes, the adoption of a multidisciplinary approach to managing nutritional risk has gained considerable attention due to its potential to substantially improve patient outcomes and overall quality of life (QoL) and reduce healthcare costs. However, the question remains: can we truly sustain such a model within the constraints of our healthcare systems?

In-hospital nutritional care is an increasingly important aspect to be managed since nutritional deficiencies have been associated with several chronic and acute pathological conditions requiring hospitalization [1,2,3]. Disease-related malnutrition has been recognized as a frequent and impactful problem in all clinical settings, particularly among the growing population of elderly patients [4]. Furthermore, the prevalence and severity of malnutrition are very high in oncological, gastrointestinal, and neurological diseases [5]. In-hospital malnutrition is a widespread problem, with prevalence estimates ranging from 20 to 50% [6,7].

Key indicators of malnutrition are unintentional weight loss and impaired body composition, mirrored by the depletion of muscle mass, fat stores, and essential body proteins, often resulting from insufficient food intake or increased metabolic demand [8]. One of the most concerning aspects of these changes is sarcopenia, a condition defined by the progressive loss of skeletal muscle mass and function [9]. It is recognized as a predictor of adverse outcomes, including increased rates of re-hospitalization and mortality [10,11,12,13]. Several studies have shown that sarcopenia is a strong predictor of decreased progression-free survival and disease-free survival, particularly in cancer patients, where the ability to cope with the disease and treatment is compromised by muscle loss [14,15,16,17].

In the pediatric setting, malnutrition can be even more harmful, as it affects the acquisition of developmental stages and prevents children from reaching their physical and cognitive potential [18,19]. Indeed, in children, the assessment of body size, composition, and growth rate are fundamental to frame their nutritional status. Serial measurements of weight and height are advisable for determining malnutrition since growth faltering is likely the best indicator of low nutritional status. Compared to adults, in growing children, not only weight loss but also absent or slow weight gain may indicate undernutrition [20].

This weakened state, both in adults and children, has a major prognostic impact by affecting the body’s ability to withstand and respond to medical treatment [21]. Additionally, malnutrition contributes to a range of complications that further prolong recovery, such as the increased complication rate, impaired wound healing, and susceptibility to infections [22,23]. As a result, malnourished patients often have significantly longer hospitalizations [23,24,25,26].

Prolonged hospital stay not only affects patients but also imposes a significant burden on healthcare systems and increases healthcare costs. Allard et al. [27] reported that malnutrition contributes to 50–75% higher healthcare costs for hospitalized malnourished patients compared to well-nourished patients. In Europe, malnutrition adds between EUR 1640 and EUR 5829 in costs per hospitalized patient due to prolonged stays, increased complications, and increased readmissions [28]. In Italy, it is estimated that healthcare providers face nearly USD 12 billion in additional annual costs due to the longer length of stay (LOS) associated with malnutrition-related disorders [29].

Despite the evidence provided by the international literature and the availability of updated international guidelines for nutritional care in several clinical settings [30], such as oncology [31,32], many patients still do not receive adequate nutritional support [5,33,34]. This might be related to the lack of (i) awareness of nutritional issues among healthcare professionals, (ii) structured collaboration between physicians and clinical nutrition specialists/dietitians, and (iii) evidence supporting the notion that nutritional support improves clinically relevant outcomes measures beyond nutritional parameters.

Addressing nutritional risk through a multidisciplinary approach has become a key determinant in enhancing the quality of patient care.

Ensuring the sustainability and the implementation of this multidisciplinary approach involves overcoming financial and logistical barriers. In the Lombardy Region (northern Italy), several initiatives, such as the establishment of the Clinical Nutrition Network of Lombardy (Italy), reported in the present paper, have made strides in improving multidisciplinary nutritional care by promoting scientific networking and standardized practices across hospitals.

## 2. Nutritional Screening

The high prevalence of malnutrition in hospitalized patients underlines the need for robust screening protocols to guide patient care and resource allocation [35]. Early detection of malnutrition is essential to implement effective interventions [36] and should be performed with validated screening tools that consider the peculiarities of the age groups. In the pediatric setting, several screening tools with different indicators, reference ages, and peculiarities have been validated in populations of hospitalized children [37,38,39,40,41,42]. Compared with other nutritional screening tools, the Screening Tool for Risk On Nutritional status and Growth (STRONGkids), being administered in all pediatric age groups from 1 month to 18 years of age, has gained considerable attention for its higher reproducibility and predictive capacity [43]. This tool has been validated in several European pediatric populations, including Italy [44]. Instead, in adult patients, the Nutritional Risk Screening 2002 (NRS-2002) and the Malnutrition Universal Screening Tool (MUST) are the most suitable [45] and validated against reference standards in different in-hospital populations compared to other screening tools [46]. Hence, we chose these tools due to their validity and widespread use in clinical and research settings.

Different disease types, such as gastrointestinal and neurological diseases, may require additional screening tools to complement the identification of nutritional risk. For example, in gastrointestinal diseases, the identification of malabsorption through specific screening tools that assess nutrient absorption and digestive functions might be of added value [47]. Similarly, in neurological diseases, the presence of dysphagia, as well as physical and cognitive impairments, may require supplementary tools to better assess the risk of malnutrition and tailor interventions accordingly [48].

Nutritional risk screening is a simple and rapid method to identify patients at risk of malnutrition, thus enabling timely support. The added value of systematic nutritional risk screening upon hospital admission is evident, as it allows for the prevention of the escalation of malnutrition-related complications [49]. This, in turn, reduces LOS, contains healthcare costs, and contributes to improving and preserving patients’ QoL.

Effective nutritional risk management requires a multidisciplinary approach [50]. The fight against malnutrition goes beyond medical treatment to encompass the holistic well-being of patients. It combines the expertise of dietitians, physicians specialized in clinical nutrition, pharmacists, nurses, and other healthcare professionals (e.g., physiotherapists) to address all patients’ needs. This collaborative approach ensures that nutritional care is not only evidence-based but also tailored to the individual needs of each patient, with the aim of minimizing treatment-related side effects and enhancing recovery and resilience to stressful events during their treatment journey [51].

## 3. Current Challenges, Limitations, and Unmet Needs

Despite the increasing recognition of malnutrition as a critical factor in patient outcomes, several barriers hinder its effective prevention, detection, and management.

A major issue is the inconsistent use of nutritional screening tools across hospitals. Although validated tools are widely available [52], their application is not standardized, with many hospitals failing to implement routine screenings upon admission, during hospitalization, or at discharge [53,54,55,56,57].

Even when malnutrition is identified, advanced nutritional interventions, such as enteral or parenteral feeding, are often delayed or avoided due to concerns about potential complications, costs, or lack of specific skills [58]. Additionally, oral nutritional supplements are often under-prescribed or not provided at all [59,60,61,62]. Ensuring adequate staff training concerning these topics is essential to increase awareness among healthcare professionals and promote timely initiation of nutritional support [57].

The fragmented regional and international policies concerning nutritional support reimbursement further worsen this scenario. Specifically, in the Italian context, although enteral and parenteral nutrition are freely provided in both in-hospital and community settings, the free reimbursement of oral nutritional supplements is limited to the inpatient setting, leaving the choice of reimbursement for the outpatient setting up to each region [63].

The infrastructure to support comprehensive nutritional programs is insufficient in many healthcare settings [64,65,66], particularly those with limited resources [67]. A shortage of qualified healthcare professionals, especially in clinical nutrition [68], can lead to gaps in patient care, making it difficult to address the complex nature of malnutrition [69].

Additionally, effective professional coordination may be a logistical barrier, as it is often difficult to ensure that all team members (nurses, physicians/pediatricians, dietitians, pharmacists, and other healthcare professionals) operate in a coordinated manner, share information efficiently, and align treatment strategies. Obstacles that may arise include differences in communication strategies, conflicting priorities, disparities in knowledge and expertise, and time constraints that hinder collaboration. Implementing clear communication strategies, fostering mutual respect, and promoting regular cross-disciplinary training are key measures to address these obstacles.

Technology integration offers promising solutions.

Electronic health record systems, telemedicine, and digital health tools are valuable allies in improving communication and data sharing, thus ensuring that all members of multidisciplinary teams have access to real-time patient data [70]. Also, AI-driven nutritional assessments are time-efficient and user-friendly tools that might improve the quality and sustainability of the multidisciplinary nutritional care process [71]. Beyond these aspects, technology integration rises as an appealing solution for tools development to early detect malnutrition risk [72,73].

However, the implementation of such systems requires significant investments in infrastructure and training, as well as overcoming ethical concerns related to data privacy, security, and consent [74]. In our time, where cyber-attacks are increasingly common, protecting patients’ privacy through robust security measures is essential [75]. In addition, patients’ willingness to share personal health data, a cornerstone of ethical healthcare, is becoming a challenging aspect to manage with the advent of technology [76].

Even though early nutritional support, specialized staff, and technology might represent a financial challenge in a resource-constrained healthcare system, this approach is ultimately a more cost-effective intervention with a significantly lower financial burden than untreated malnutrition [77,78].

The effectiveness of a multidisciplinary approach in constraining costs and improving patients’ outcomes is supported by a great number of studies [50,79,80,81,82,83,84]. Hassel J.T. et al. reported that for each USD 1 invested in the multidisciplinary nutritional team, a saving of USD 4.20 is realized. This was paralleled by a numerical but not significant reduction in mortality rate, LOS, and readmission rate [85]. The multidisciplinary nutritional approach effectively improved nutritional status parameters, reduced malnutrition prevalence, and constrained the decline in quality of life among older adults with hip fractures [83]. Moreover, introducing a multidisciplinary nutritional approach effectively increases dietary intake and LOS and reduces the cost associated with the use of parenteral nutrition among adult patients in intensive care units [82,84]. These results were also confirmed among pediatric patients [86].

The long-term sustainability of a multidisciplinary model to manage in-hospital malnutrition is not the responsibility of healthcare providers alone; it requires a concerted effort by governments, institutions, healthcare authorities, and organizations to allocate resources, promote education, and establish evidence-based guidelines.

## 4. The Multidisciplinary Nutritional Team in the Italian Context

In some counties, dietitians have more autonomy, as they can prescribe without any supervision oral nutritional supplements and enteral or parenteral nutrition [33]. However, they are always part of a multidisciplinary team and actively collaborate with physicians.

In Italy, there is a peculiar structure for nutritional risk management. Hospital dietitians work under medical supervision; they estimate patients’ nutritional requirements to provide appropriate nutritional counseling and support/monitor patients’ nutritional interventions prescribed by physicians, thus improving patients’ clinical outcomes [87,88].

This professional interaction is based on close teamwork with physicians, where dietitians actively contribute to ensure that nutritional strategies are well integrated with the overall therapeutic plan.

In addition, dietitians play a pivotal role in managing the nutrition-impact symptoms (e.g., nausea, loss of appetite, and taste alterations), which can further worsen patients’ nutritional status [89].

Unfortunately, dietitians are one of the most understaffed professions in Italy, making the early detection and initial management of malnutrition by them alone challenging or even impracticable. In fact, clinical nutrition units in Italy are few, and even fewer can rely on in-house nurses or support staff to assist dietitians in the early phases of malnutrition risk identification and the nutritional care process [90]. Ward nurses and support staff are usually the first health professionals to admit patients to the hospital and could therefore screen them for nutritional risk and provide oral nutritional supplements (ONS) as appropriate. Nurses could also supervise the assessment of food intake and the monitoring of nutritional status during hospital stay [91], allowing dietitians and physicians to provide timely and appropriate nutritional support. They could also translate complex nutritional information into easy-to-understand language to help patients understand the importance of maintaining adequate nutritional status. Nurses, despite their role in screening and monitoring, are not expected to diagnose or intervene in complex cases of malnutrition. The shared responsibility between dietitians and nurses ensures that while dietitians can focus on complex diagnoses and tailored interventions, nurses are instrumental in the early identification of nutritional risk and supporting basic nutritional care.

The multidisciplinary nutritional risk management should also include physiotherapists. Their expertise can significantly contribute, in conjunction with appropriate nutritional support, to preventing the loss and reinforcing the gain of muscle mass during hospitalization [92].

A multidisciplinary approach to in-hospital nutritional risk foresees the collaboration among several professional figures with the common goal of patient’s well-being, promoting patients empowerment to take an active role in their own care, fostering a sense of control and positivity during a challenging time. In resource-constrained healthcare settings, the contribution of nurses with appropriate clinical nutrition training to support nutritional management provision might be essential.

All these observations were the basis for the proposal and development of the algorithm for nutritional risk management of the Clinical Nutrition Network of Lombardy.

## 5. The Clinical Nutrition Network of Lombardy

Since October 2021, the Lombardy Region has supported the significant growth of clinical nutrition. Prior to this, the only available regional resolution on home artificial nutrition (HAN) was dated 1992 [93]. This document was general, outlining only the basic aspects of HAN provision. On 25 October 2021, the Lombardy Region approved the Shared Care Plan on Home Artificial Nutrition (Welfare Decree n° 14274, 25 October 2021) [94], which provides a detailed and comprehensive guide for organizational and technical support in managing patients requiring HAN. In addition, the decree led to the establishment of multidisciplinary nutritional teams (MNTs) in hospitals lacking structured clinical nutrition units (CNUs) [95]. Furthermore, Lombardy became the first region in Italy to make nutritional screening mandatory with Resolution n° XII/1812, 29 January 2024 [96]. This decree aims to ensure that all hospitalized patients and those receiving home care undergo a malnutrition risk assessment upon hospitalization and discharge, which has the potential to significantly improve the quality of patient care, reduce long-term healthcare costs, and raise public awareness of the importance of proper nutritional care. However, to achieve these benefits, it will be crucial to ensure effective implementation and overcome organizational challenges. A key element in supporting these initiatives is the Regional Technical Table for Nutrition Safety (Tavolo Regionale di Sicurezza Nazionale—TARSIN), which involves the local healthcare authorities throughout the region [97].

In addition to these important achievements, the Clinical Nutrition Network of Lombardy (Rete della Nutrizione Clinica Lombarda) was established on 18 October 2022, with the broad objective of overcoming the inequalities in the management of nutritional care by promoting clinical–scientific networking, thus sharing the best nutritional clinical practices among all the regional hospitals [98].

The main objectives of the Clinical Nutrition Network of Lombardy are as follows:

Implementation of a regional Clinical Nutrition Network involving all the regional CNUs focused on the improvement of nutritional care practices in synergy with prevention and health promotion services.

1. Implementation of the Welfare Decree n° 14274 of 25 October 2021 on the management of HAN;
2. Revision of the allocation of resources and staff dedicated to clinical nutrition within the Regional Health Service (RHS);
3. Formulation and implementation of a tariff system for clinical nutrition services;
4. Introduction of mandatory nutritional screening in all RHS structures;
5. Establishment of a recognized and accountable telemedicine system for monitoring patients undergoing HAN and bariatric surgery;
6. Implementation of a digital platform for HAN prescription and management;
7. Development of structured collaborations with other regional clinical networks, such as those for Anesthesia, Oncology, Hepato-Gastroenterology, Endocrinology–Diabetology, Neuroscience, Rare Disease, Surgery, Internal Medicine, and Mental Health;
8. Development of hub-and-spoke pathways for specific relevant pathological conditions, including cancer (particularly in the context of specific disease units, such as for pancreatic and lung cancer), benign chronic intestinal failure, eating disorders, and grade III obesity;
9. Establishment of a regional clinical nutrition education program, primarily for MNT members, in close collaboration with the regional Directorate for Training and Employment.

## 6. Proposal of a New Multidisciplinary Approach to Nutritional Risk Management

Growing recognition of the impact of malnutrition has led to a shift toward more comprehensive, multidisciplinary approaches to nutritional care. A more integrated model is needed to prioritize collaboration across specialties (dietitians, physicians, nurses, and other specialties), adopt standardized nutritional screening and support practices, and leverage new technologies to improve clinical monitoring, ensure continuity of care, and address the diverse aspects of malnutrition, thus successfully managing in-hospital nutritional risk.

**Figure 1:**
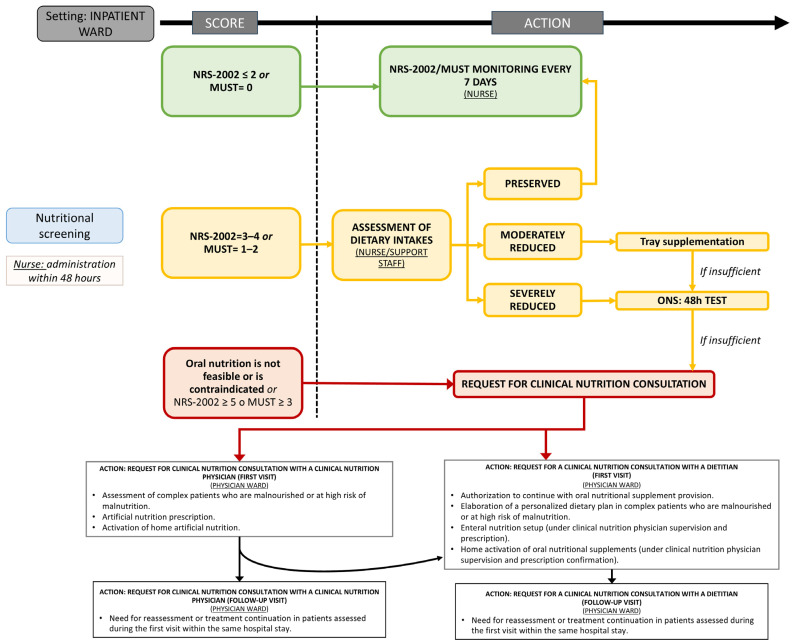
Algorithm for malnutrition risk management in adults proposed by the Clinical Nutrition Network of Lombardy.

**Figure 2:**
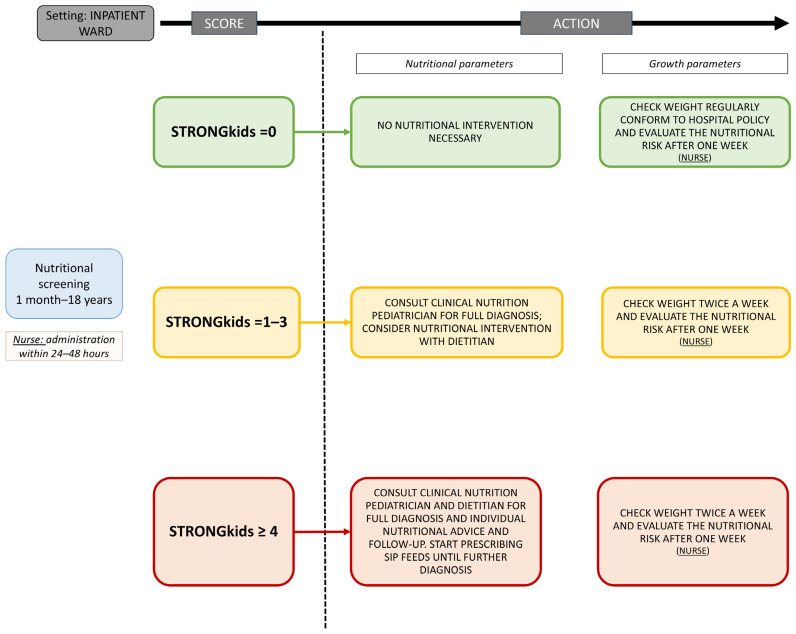
Algorithm for malnutrition risk management in children proposed by the Clinical Nutrition Network of Lombardy.

Early identification of malnutrition through routine screening at admission, during hospitalization, and at discharge helps ensure that patients at risk are flagged and receive timely intervention. While implementing such a comprehensive model requires an initial investment in training, resources, and technology, the long-term benefits are substantial. By reducing the incidence of malnutrition-related complications, shortening hospital stays, and lowering readmission rates, healthcare systems can achieve significant cost savings while improving clinical outcomes. Specifically, the Clinical Nutrition Network of Lombardy has proposed a new diagnostic and therapeutic algorithm for the management of malnutrition risk, which implies the early systematic involvement of nurses, summarized in Figure 1 and Figure 2. In adults, nutritional risk screening should be systematically performed within 24–48 h of hospital admission, ensuring prompt intervention to prevent further nutritional deterioration. Properly trained nurses should be responsible for nutritional risk screening as they are the first healthcare figures who accommodate patients. Subsequently, nutritional management should be based on nutritional risk scores.

Patients identified with scores of NRS-2002 ≤ 2 or MUST = 0 should be monitored by systematic reassessment approximately every 7 days. Patients who achieve scores of NRS-2002 = 3–4 or MUST = 1–2 should have dietary intake assessed and monitored by ward nurses or support staff using specifically designed food consumption record forms.

If dietary intake is preserved, nutritional risk evaluation should continue on a weekly basis. Regular monitoring and weekly assessment reduce the risk of undetected deterioration of nutritional status during hospitalization.

If nutritional intake is moderately reduced, nurses should promptly initiate a tray supplementation with snacks or additional food, according to patient preferences and clinical conditions.

If this is insufficient to guarantee adequate protein-calorie consumption, or if patients present with severely reduced nutritional intakes, ONS should be provided for a 48 h test upon request by the ward physicians, followed by a request for clinical nutrition consultation.

If patients with scores of NRS ≥ 5 or MUST ≥ 3 are identified, or if oral nutrition is not feasible or contraindicated, specialized medical nutritional support consultation should be requested. In this context, adequate training of nursing and support staff is crucial, as well as efficient allocation of resources for nutritional monitoring, supplementation, and clinical nutrition consultation. This approach can allow dietitians and clinical nutrition physicians to provide timely and appropriate nutritional support while ensuring early food integration or ONS provision without avoidable delays.

Ward physicians should carefully evaluate the type of nutritional consultation needed for each patient. Specifically, a consultation with a clinical nutrition physician may be appropriate for (i) assessment of complex patients who are malnourished or at high risk of malnutrition, (ii) artificial nutrition prescription, and (iii) activation of home artificial nutrition. Instead, a dietitian consultation may be indicated for (i) authorization to continue with ONS provision during the hospital stay, (ii) elaboration of a personalized dietary plan for complex patients who are malnourished or at high risk of malnutrition, (iii) enteral nutrition setup (under the supervision and prescription of a clinical nutrition physician), and (iv) activation of home ONS provision (under the supervision and prescription of a clinical nutrition physician) (Figure 1).

For pediatric patients, the STRONGkids questionnaire can be performed directly upon admission and does not require trained personnel: written instructions alone allow health professionals (such as nurses) to complete the questionnaires. Briefly, those patients with a STRONGkids score = 0 need to be monitored approximately every 7 days for nutritional screening, and their weight should be assessed regularly as a growth parameter. Instead, for those patients identified with a STRONGkids score = 1–3, a consult with a clinical nutrition physician should be requested for full diagnosis, and a nutritional intervention with a dietitian should be considered; their weight should be assessed twice a week. Finally, for those patients identified with a STRONGkids score ≥ 4, a consult with a clinical nutrition physician and a nutritional intervention with a dietitian should be requested; their weight should be assessed twice a week (Figure 2).

## 7. Discussion

The activities and the in-hospital multidisciplinary approach proposed by the Clinical Nutrition Network of Lombardy were built considering the available literature and the clinical experience of field specialists. However, the real-world effectiveness of these strategies, once implemented, will need to be assessed through tailored clinical trials, incorporating appropriate comparative analysis and cost-effectiveness evaluations, including the cost of model implementation and well-defined process and outcomes indicators.

Beyond providing insight into the effectiveness of the model, these evaluations will help define key aspects of its sustainability, which will need to be further explored through additional criteria, such as long-term feasibility and integration into existing clinical workflow. Undoubtedly, the current lack of real-world data is a limitation, but addressing this gap is already part of the planned activities of the Clinical Nutrition Network of Lombardy.

While the general principles of nutritional risk management are shared, the approach to care differs between adult and pediatric settings. In pediatrics, greater emphasis is placed on growth monitoring, developmental outcomes, and family involvement, whereas adult care focuses more on managing comorbidities and functional decline. These differences influence how nutritional screening and interventions are prioritized and implemented. For these reasons, two distinct algorithms have been proposed, each tailored to the specific clinical and organizational needs of the respective populations.

The herein proposed multidisciplinary approach was designed considering mainly the regional context; however, its pillars might be easily extended to other countries and regions, as the pertinent national and international guidelines are easily available, and no relevant additional costs are needed to implement them. In resource-poor areas, challenges such as insufficient access to trained healthcare professionals, inadequate technological infrastructures, and budget constraints may impede the full implementation of the model. Additionally, the variability in healthcare systems across different regions could affect the model’s applicability. These challenges might be effectively addressed by the employment of simple screening tools, the involvement of other professional figures (e.g., nurses), and the promotion of the local healthcare network.

The achievement of a multidisciplinary in-hospital nutritional risk management, including mandatory risk screening at hospital admission, continuous monitoring of nutritional status, and the administration of nutritional interventions, might be achieved through pragmatic and strict healthcare policies (e.g., laws and regulations) based on each region/country organization and asset.

Dietitians, clinical nutrition physicians, ward physicians, pharmacists, and nurses together form a holistic task force that can optimize both the clinical and economic aspects of nutritional care.

While several challenges persist, the value of this approach should be accurately considered by Healthcare Authorities and Institutions. By capitalizing on the unique contributions of each professional and leveraging technology, the creation of sustainable models that place the holistic well-being of patients at the center of healthcare management should be strongly promoted.

The Clinical Nutrition Network of Lombardy will continue its activities with the aim of implementing these models and contributing to improving the quality of healthcare by integrating clinical nutrition into the global management of patients’ needs.

The next steps should focus on several key areas to be improved and/or implemented to overcome the current barriers and limitations, thus moving toward the multidisciplinary management of nutritional risk. Stringent regulations, incentives, and sanctions are crucial to compel Hospital Directions to systematically implement nutritional screening (application of Resolution n° XII/1812, 29 January 2024) and early nutritional support. This would also promote a more efficient allocation of resources and staff, directing them toward patients needing nutritional consultation. Developing and implementing a tariff system for clinical nutrition will ensure appropriate resource allocation.

Including nurses and auxiliary staff as a key component of clinical nutrition units and promoting a multidisciplinary approach will also contribute to more effective resource use and will be fundamental to addressing the significant challenge of a limited workforce.

Beyond all these aspects, it is equally important to deliver proper education to multidisciplinary nutritional team members, especially nurses, providing basic skills in malnutrition management and raising awareness of the importance of nutritional care. Implementing clinical nutrition classes in university courses and creating novel opportunities for continuous professional training are strategies that might optimize the delivery of clinical nutrition services and efficient resource use.

The use of technology will also play a key role in improving healthcare practices. Introducing a digital platform for uniform prescription and management of HAN in the whole regional territory will enhance communication, facilitate data sharing, and streamline procedures.

These steps are at the core of the activities of the Clinical Nutrition Network of Lombardy.

By implementing these strategies, the network aims to optimize both the clinical and economic aspects of nutritional care, ensuring better patient outcomes and more efficient healthcare delivery.

The potential benefits of multidisciplinary nutritional risk management are substantial. Governments should prioritize healthcare funding and policy reforms that support multidisciplinary care models and address systemic challenges such as workforce shortages. Healthcare organizations should create environments where teamwork and collaboration are integral to daily activities, while researchers play a critical role in generating evidence that demonstrates the efficacy and cost-effectiveness of these models.

Therefore, policymakers, healthcare leaders, and researchers should all cooperate to overcome barriers and address challenges to effectively implement and ensure the sustainability of multidisciplinary nutritional risk management as in-hospital standard nutritional care.
